# Toxic heavy metal exposure and heart health: a systematic review and meta-analysis of 324,331 patients

**DOI:** 10.1186/s12872-025-05248-9

**Published:** 2025-11-07

**Authors:** Shamikha Cheema, Syed Ibad Hussain, Muhammad Shaheer Bin Faheem, Amna Amir Jalal, Mohamed Rifai, Areej Dar, Muhammad Burhan, Areeba Shahid, Muhammad Seerat Ali, Amna Anwar, Misha Khalid, Sumaya Samadi

**Affiliations:** 1 King Edward Medical University, Lahore, Pakistan; 2 Jinnah Sindh Medical University, JSMU, Karachi, Pakistan; 3 Department of Medicine and Surgery, Karachi Institute of Medical Sciences, KIMS, Karachi, Pakistan; 4 Menoufia University, Shebin El Kom, Egypt; 5 Shaikh Khalifa Bin Zayed Al Nahyan Medical College, Lahore, Pakistan; 6 Dow University of Heath Sciences, Karachi, Pakistan; 7 Quaid-E-Azam Medical College, Lahore, Pakistan; 8 Federal Medical and Dental College, FMDC, Islamabad, Pakistan; 9 Karachi Medical and Dental College, Karachi, Pakistan; 10 Kabul University of Medical Sciences “Abu Ali Ibn Sina”, Kabul, Afghanistan

**Keywords:** Toxic Heavy Metal Exposure, Heart Health, Meta-analysis, Cardiovascular diseases

## Abstract

**Introduction:**

Heavy metal pollution is a concerning cardiovascular risk factor and a major public health concern. While traditional cardiovascular disease (CVD) risk factors are well-established, environmental exposures are less recognized, despite their growing significance.

**Methods:**

Adhering to PROSPERO and PRISMA guidelines, a search was conducted across PubMed, Cochrane, and Embase until April 2025 for studies involving patients with heart disease or stroke who were exposed to heavy metals. Primary outcomes were the events of CVD, CHD, and stroke. Proportions of events within populations with 95% confidence intervals were pooled. R software was used for the analysis, with statistical significance set at *p* < 0.05.

**Results:**

We included 16 studies involving 324,331 participants. The cadmium subgroup showed a pooled proportion of 0.09 (95% CI: 0.01–0.17), with individual values ranging from 0.01 to 0.22; the arsenic subgroup showed a pooled proportion of 0.04 (95% CI: 0.04–0.04). The overall pooled proportion was 0.08 (95% CI: 0.01–0.15). Six studies found cases of CHD in groups exposed to cadmium and arsenic, with combined rates of 0.10 (95% CI: 0.00–0.22) for cadmium and 0.00 (95% CI: 0.00–0.00) for arsenic. The overall pooled proportion was 0.08 (95% CI: 0.00–0.18). Ten studies assessed CVD proportions by metal exposure. Arsenic showed a pooled proportion of 0.06 (95% CI: 0.00–0.18), cadmium 0.14 (95% CI: 0.00–0.31), lead 0.08 (95% CI: 0.01–0.14), and mercury 0.05 (95% CI: 0.04–0.06). The overall pooled proportion was 0.10 (95% CI: 0.03–0.17).

**Conclusion:**

This study emphasizes the significance of the toxic environmental metals contributing to cardiovascular risk beyond behavioural risk factors.

**Supplementary Information:**

The online version contains supplementary material available at 10.1186/s12872-025-05248-9.

## Introduction

Environmental pollution is one of the most pressing problems of the twenty-first century. Among pollutants, heavy metals such as cadmium, arsenic, mercury, copper, and lead are particularly disturbing due to their high presence in the environment and the damage they cause to the human body [1, 2]. These metals infiltrate water sources, soil, and food after prolonged exposure and easily get into our daily lives [3]. Cardiovascular diseases, which are the leading cause of death globally, accounting for more than 17 million deaths annually, are strongly associated with heavy metals [4]. Classic risk factors, including hypertension, dyslipidemia, smoking, and diabetes, have been extensively investigated. Environmental exposures to contaminants such as arsenic, cadmium, and mercury are now acknowledged to have significant contributions to CVD risk [5]. These heavy metals disrupt CVS functions by chronic inflammation, oxidation, endothelial dysfunction, and disturbances in lipid metabolism [4].

Extensive research has established strong relationships between chronic exposure to heavy metals and elevated risks for CVD. WHO estimates that more than 200 million people are chronically exposed to inorganic arsenic (As) contaminated groundwater, mainly affecting poor countries [2, 3]. Chronic exposure to arsenic, cadmium, and mercury has become known as an unrecognized contaminant of cardiovascular health, as these heavy metals are widely distributed in the environment through both natural processes and anthropogenic activities. Furthermore, recent research has also shown an association between adult CVD risk and lead exposure. It was discovered that low exposure levels to lead were positively connected with cardiovascular disease and cardiovascular risk factors [5].

The first comprehensive meta-analysis was carried out in 2018 by Chowdhury et al. to study the quantitative relationship between environmental toxic metal contaminants and the risk of cardiovascular disease. The study results show an association between arsenic, lead, cadmium, and copper with elevated CVD risk (relative risks: 1.23–2.22), while mercury showed no significant link with CVD. Strengths of the study included individual-level exposure assessment and dose–response analyses. However, considerable gaps remain in understanding the entire extent of these dangers. Most studies have concentrated on high-income regions, ignoring low- and middle-income nations, where industrial activity and loose regulations increase exposure. Interactions between metal combinations, such as arsenic-cadmium co-exposure, are understudied despite their real-world occurrence, and new contaminants, such as cobalt and nickel, lack extensive epidemiologic data [6].

Now, the urgency to address these gaps is underscored by the advancement in literature, considering several new studies have been conducted since 2018 with enhanced exposure measures, such as policy shifts in the US funding lead pipe replacement and EU restrictions on cadmium fertilizers. These trends underline the necessity for an up-to-date, internationally inclusive evidence foundation to guide equitable public health measures [7].

Our analysis adds data from recent studies to quantify the links between heavy metals and CVD, investigate dose–response relationships and metal interactions, and examine geographic, genetic, and sex-based variability. This study intends to promote the global prevention of cardiovascular disease and bridge gaps in environmental health research by including underrepresented regions and explaining findings within modern policy frameworks.

## Methods

This meta-analysis was reported following the Preferred Reporting Items for Systematic Reviews and meta-analyses (PRISMA) guidelines [8]. The protocol of this meta-analysis was registered in the International Prospective Register of Systematic Reviews (PROSPERO) with the reference number CRD420251020688.

### Eligibility criteria

#### Inclusion criteria

We included 1) observational studies, including the adult population (18 + years) with heart disease, and 2) populations exposed to either a single heavy metal or multiple heavy metals, including cadmium, mercury, arsenic, or lead. 3) Peer-reviewed articles published in the English language, 4) Studies reporting quantitative measures of association for any cardiovascular outcomes—such as stroke, cardiovascular disease (CVD), or coronary artery disease (CAD)—were included, even if only a single outcome was reported.

#### Exclusion criteria

Studies on animals, case reports, abstracts, and studies with no poolable cardiovascular outcomes were excluded.

#### Data sources and search strategy

A thorough search was carried out using the Cochrane CENTRAL, PubMed/MEDLINE, and Google Scholar databases. The reference lists of the retrieved publications, as well as previous meta-analyses, were examined for relevant articles. The search method placed no constraints on publishing status or language. The search words included relevant PubMed.

MeSH terms and associated keywords, such as (Cardiology, Cardiovascular Diseases, Coronary Artery Disease, Heart Diseases, Heart Failure, Environmental Exposure, Environmental Medicine, Environmental pollutants, Mercury, Metals, Chromium, Copper, Nickel. The detailed search strategy is provided in Supplementary Table [S1].

#### Study selection

All articles retrieved through the systematic search were imported into the EndNote reference library, version X8.1 (Clarivate Analytics), where duplicates were thereafter omitted. Two authors independently examined and selected studies, and any discrepancies were resolved by a third author. Selected studies were retrieved for full-text review to ensure relevance. The inclusion criteria were defined using the Population, Intervention, Control, and Results (PICO) methodology for systematic reviews, where P denotes trials assessing patients with Heart disease, I denotes Heavy Metals, C denotes placebo, and O denotes safety and efficacy outcomes.

### Data extraction

On a pre-piloted Microsoft Excel sheet, two authors independently evaluated the data and supplemental resources; disagreements were settled by consulting a third author. From the selected studies, the following information was extracted: study labels, year of publication, study design, baseline patient characteristics, weight, BMI, and length of illness, as well as results about the heavy metals exposure, safety, and effectiveness profile.

### Quality assessment

Two authors analyzed the quality of included observational studies using the Newcastle–Ottawa Scale/JBI Critical Appraisal Checklist. This tool assesses the risk of bias associated with patient selection, study performance, outcome detection, data attrition, study findings reporting, and other types of bias. The judgments can be 'Low' or 'High' in terms of bias risk, or they can suggest 'Some concerns.'

#### Quality of the evidence: GRADE assessment

The quality of evidence for each outcome was assessed using the Grading of Recommendations Assessment, Development, and Evaluation (GRADE) approach, implemented through the GRADEpro Guideline Development Tool. This method classifies the certainty of evidence into four categories: high, moderate, low, or very low. High-quality evidence suggests that the true effect is close to the estimated effect, whereas very low-quality evidence indicates substantial uncertainty about the estimate. The assessment considered factors such as risk of bias, inconsistency, indirectness, imprecision, and publication bias. Judgments were made independently by two reviewers, with discrepancies resolved through a third senior reviewer.

### Statistical analysis

The study utilized R version 4.3, employing the meta and dmetar packages for statistical analysis. We used pooled proportions with a 95% CI, as most studies didn’t have a control group so RR and OR couldn’t be used. Heterogeneity was assessed according to the “Cochrane Handbook v6.3, 2022, where I2 values of 0–40% indicate low, 30–60% moderate, 50–90% substantial, and 75–100% considerable heterogeneity. A Chi-square test p-value below 0.1 was considered statistically significant for heterogeneity. Metal-outcome pairings with ≥ 3 independent studies were analyzed to avoid sparse data bias. Subgroup analyses were performed to explore potential differences according to toxic metal exposure. Leave-one-out sensitivity analyses were also conducted to assess the robustness of the findings.

Meta-regression analyses were performed using the *metainc* and *metareg* functions from the R package meta for outcomes with data available from at least 10 studies, in order to examine potential moderators of the pooled effect estimates. The moderators selected were consistently reported across the included studies and reflected participant-level characteristics that could influence risk estimates. Specifically, body mass index (BMI), age, and the male-to-female (M/F) ratio were assessed as continuous predictors of study-level variation.

## Results

### Results of literature search

**Fig. 1:**
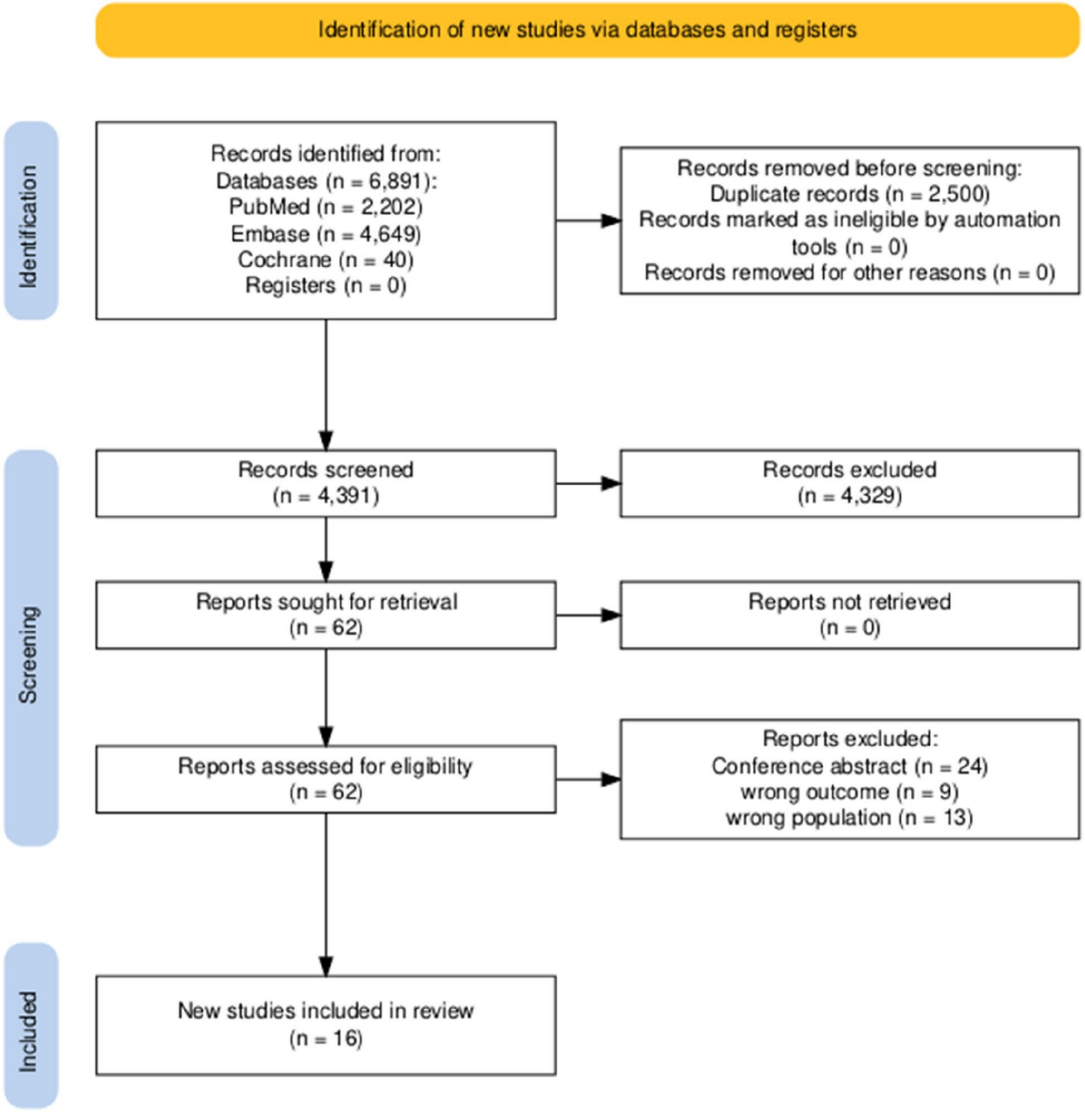
PRISMA flow diagram. *Consider, if feasible to do so, reporting the number of records identified from each database or register searched (rather than the total number across all databases/registers). **If automation tools were used, indicate how many records were excluded by a human and how many were excluded by automation tools. Source: Page MJ, et al. BMJ 2021;372:n71. https://doi.org/10.1136/bmj.n71. This work is licensed under CC BY 4.0. To view a copy of this license, visit

A systematic search was conducted across two electronic databases: PubMed (*n* = 2202), Embase (*n* = 4649), and the Cochrane Library (n = 40), yielding a total of 6891 records. After the removal of 2500 duplicate records, 4391 unique records remained for screening. A total of 62 reports were identified for full-text retrieval. Finally, all reports were successfully retrieved, with 46 reports excluded for not meeting the eligibility criteria. Ultimately, 16 studies met the inclusion criteria and were included in the final systematic review. This process is illustrated in Fig. 1, following the PRISMA 2020 flow diagram.

### Results of baseline characteristics

A total of 16 observational studies were conducted across various countries, including Bangladesh, the United States, Denmark, Sweden, Taiwan, Australia, Georgia, Korea, and Greenland. Sample sizes ranged from 200 to 98,250, and a total of (n = 324,331). Mean age ranged from as low as 17.4 years to as high as 75.2 years. Follow-up durations, where reported, spanned from 7 to 22 years. The summary of all baseline characteristics is shown (Table 1).

**Table 1:** Baseline characteristics of all studies [9–24]

Study Id	Country	Study Type	No. of Participants	Mean Age (years)	Male (%)	Total Follow-up (years)	Mean BMI	Mean Diabetes	Mean Hypertens ion	Mean Smokers	No. of Cases		
											CVD	CHD	Stroke
Arsenic													
Farzan (2022) [9]	Bangladesh	Crosssectional	200	17.4	53		20.6			2	0	0	0
Forkan (2021) [10]	Bangladesh	Crosssectional	270	49.2	74		23.65	48	48	64	0	0	0
Kuo (2021) [11]	United States	Prospective Cohort	3973	55	37	14.4	30.2	50.5	39.3	34.3	484	0	0
Annette (2018) [12]	Denmark	Cohort	53941	55	41	12.8	26	2	50	36	0	0	2195
Medgyesi (2024) [13]	United States	Prospective Cohort	98250	53	0	18.3	24	2.8	17	5.1	5313	3207	0
Lead													
Cook (2022) [14]	Georgia	Prospective Cohort	15036		48.1	22	25.9			28.8	1400	0	0
Lanphear (2018) [15]	United States	Cohort	14289	44.1	47.9	19.3	27.5	17.5	16.5	28.4	1801	988	0
Harari (2019) [16]	Sweden	Crosssectional study	4172	57	40			7.4	63	0.6	58	0	0
Lin (2020) [17]	Taiwan	Crosssectional Observational	738	21.1	39.8			1.9	8.1	16.7	0	0	0
Cadmium													
Poulsen (2021) [18]	Denmark	Case-cohort	19394	57	59	13.5	26.28				0	0	534
Sears (2022) [19]	Denmark	Case-cohort	19394	57	50	20	26.22				958	0	0
Chen (2018) [20]	United States	Case-cohort	3160	65.9	46.6	7	27.5	23	61.8	27.2	0	0	680
Deering (2018) [21]	Australia	Cohort	1359	75.2	0	14.5	27.1	6.6	56	37	529	460	207
Jeong (2020) [22]	Korea	Crosssectional	10626	40.1	50.4		23.63	5.6	18.3	27.2	80	80	60
Ma (2022) [23]	United States	Crosssectional	38223	47.22	49.6		27.6	10.5	33	25.2	0	1730	1170
Mercury													
Larsen (2018) [24]	Greenland	Prospective Cohort	3083	43.5	43.4	8	26.2			62.5	162	0	0

### Results of quality assessment

Quality assessment was performed utilizing the Newcastle–Ottawa Scale (NOS) for ten cohort and case-cohort studies, which exhibited moderate to high methodological quality, with scores ranging from 7 to 9 out of a possible 9 points. Furthermore, 6 cross-sectional studies were appraised using the Joanna Briggs Institute (JBI) checklist. All evaluated studies demonstrated a low risk of bias and robust methodological rigor. Collectively, these results indicate that the overall quality of the included studies was satisfactory**.** The results are also provided in Supplementary Material S2.

### Association of environmental contaminants with stroke

**Fig. 2:**
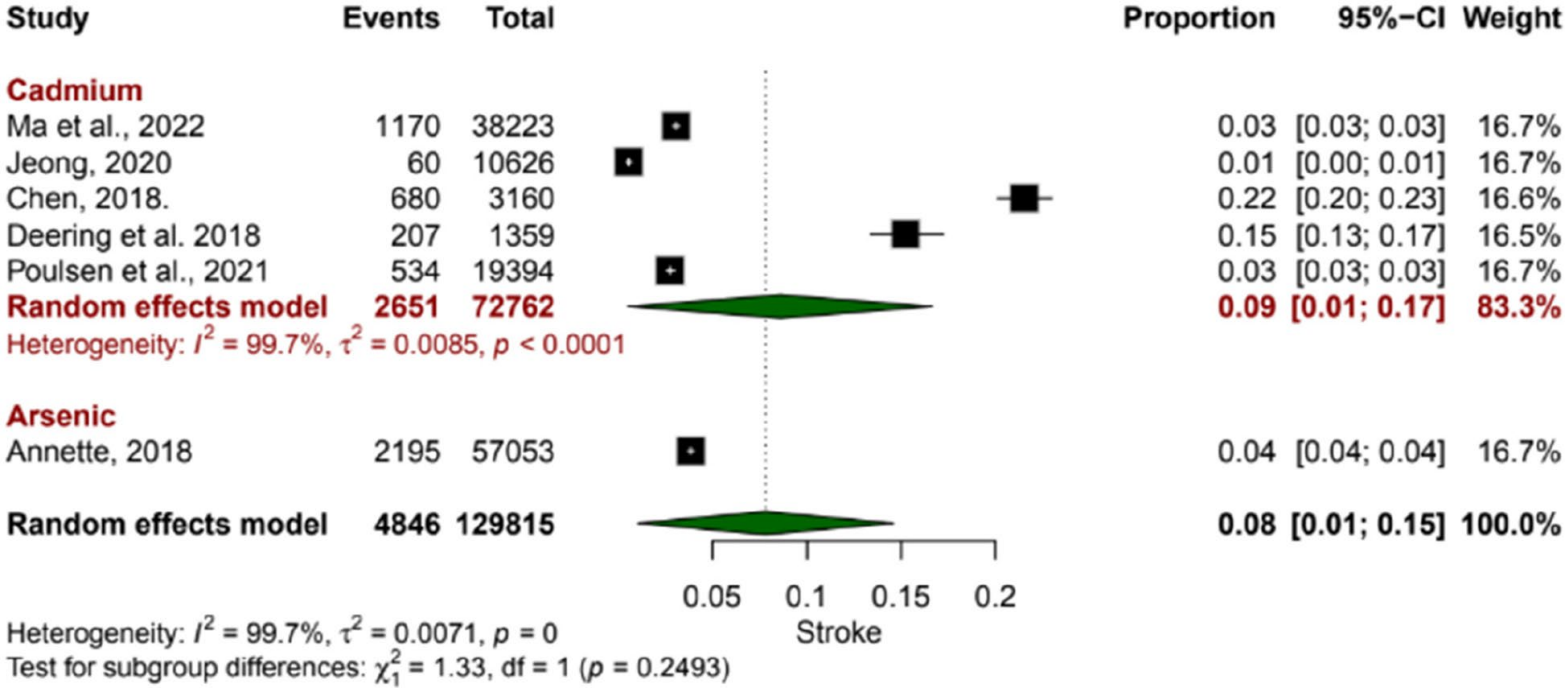
Forest plots showing the association of environmental contaminants with stroke

A total of six studies were included, assessing the proportion of stroke events among populations exposed to Cadmium and arsenic. Five studies examining the cadmium-exposed subgroup revealed a pooled proportion of 0.09 (95% CI: 0.01 to 0.17). Individual study estimates varied, with stroke proportions ranging from 0.01 to 0.22, suggesting a significant proportion of individuals to experience stroke. Similarly, one study examining the arsenic-exposed subgroup revealed a pooled proportion of 0.04 (95% CI: 0.04 to 0.04), indicating a consistently elevated proportion with less variability than the Cadmium. The overall pooled proportion was 0.08 (95% CI: 0.01 to 0.15), with substantial heterogeneity observed (I^2^ = 99.7%), indicating considerable inconsistency in effect sizes across studies (Fig. 2).

### Association of environmental contaminants with coronary heart disease

**Fig. 3:**
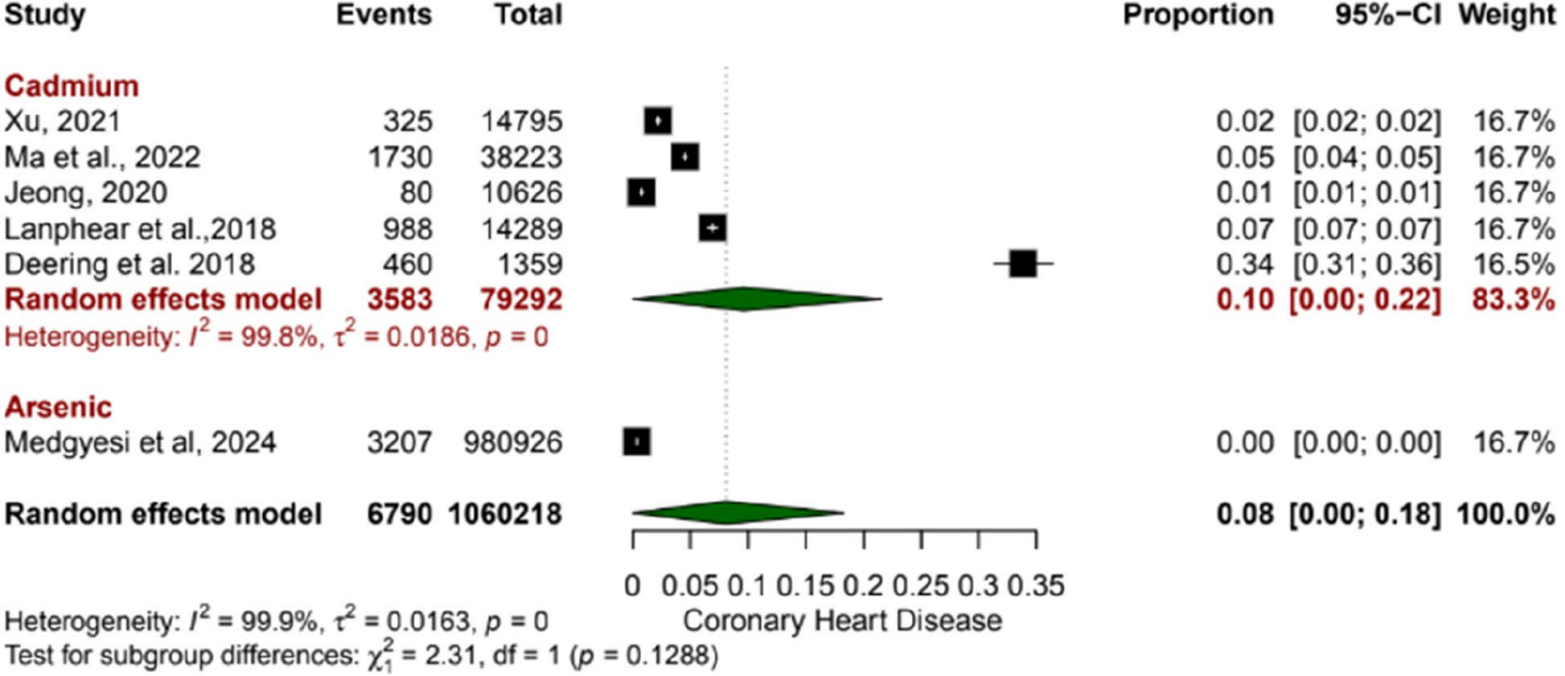
Forest plots showing the association of environmental contaminants with coronary heart disease

A total of six studies were included, out of which five studies evaluated CHD proportions among individuals exposed to cadmium, revealing a pooled proportion of 0.10 (95% CI: 0.00 to 0.22). Similarly, one study reported on CHD proportions in arsenic-exposed populations with a pooled proportion of 0.00 (95% CI: 0.00 to 0.00). The overall pooled proportion was 0.08 (95% CI: 0.00 to 0.18), suggesting 8% of individuals exposed to cadmium or arsenic may experience CHD events, with significant heterogeneity among the included studies (I^2^ = 99.9%), indicating considerable inconsistency in effect estimates (Fig. 3).

### Association of environmental Contaminants with Cardiovascular Diseases (CVD)

**Fig. 4:**
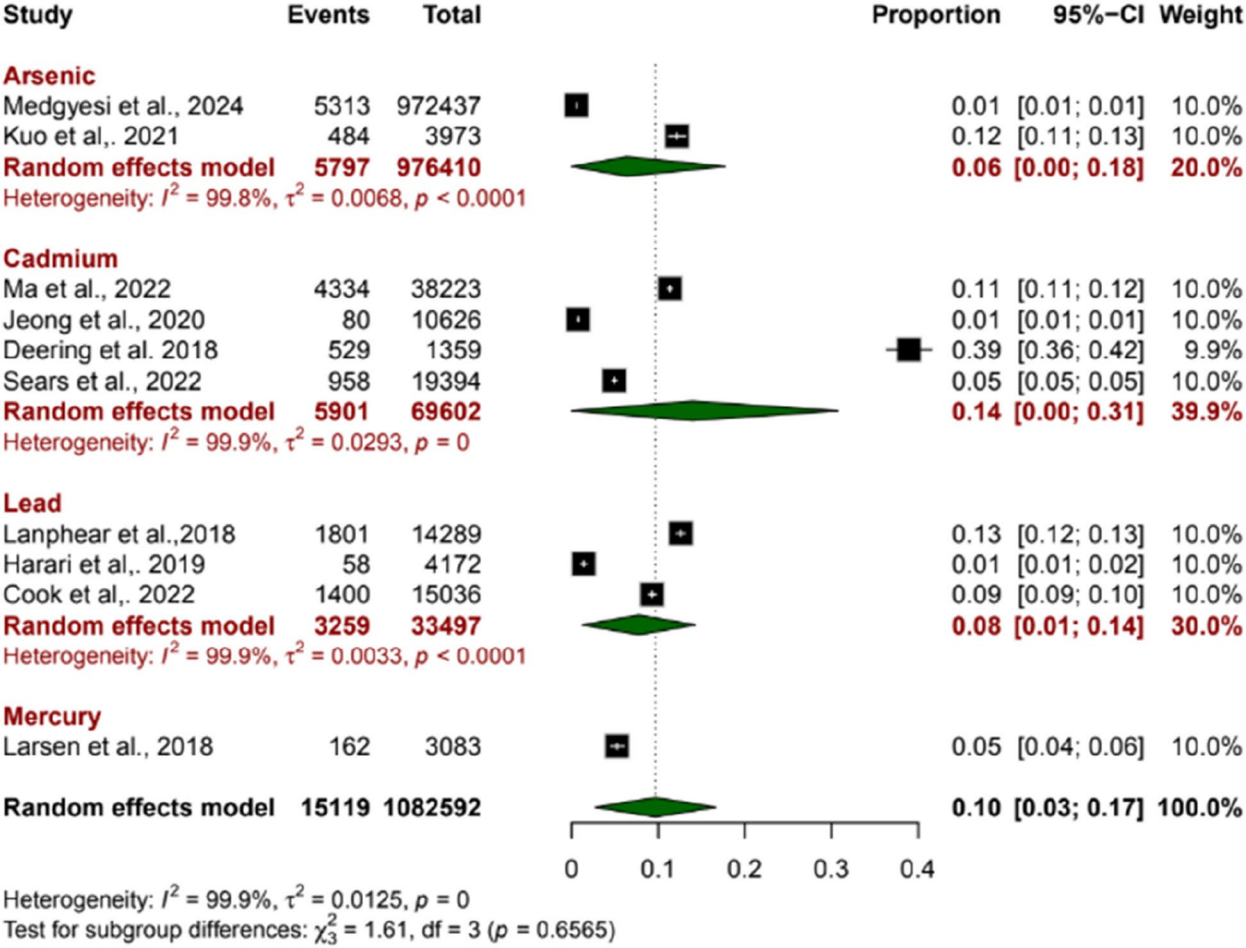
Forest plots showing the association of environmental contaminants with Cardiovascular Diseases

A total of 10 studies were included, out of which two studies analyzed the pooled proportion of (CVD related to arsenic exposure of 0.06 (95% CI: 0.00 to 0.18) and high heterogeneity (I2 = 99.8%), indicating substantial variability between studies. Additionally, for the cadmium-exposed subgroup, four studies revealed a pooled proportion of 0.14 (95% CI: 0.00 to 0.31) and high heterogeneity (I2 = 99.9%). The Lead exposed subgroup with three studies revealed a higher proportion of 0.08 (95% CI: 0.01 to 0.14) and high heterogeneity (I2 = 99.9%). Lastly, Mercury exposed subgroup analysis with only one study that revealed a proportion of 0.05 (95% CI: 0.04 to 0.06). The overall pooled proportion of CVD was revealed to be 0.10 (95% CI: 0.03–0.17), also with high heterogeneity of I2 = 99.9% (Fig. 4).

### Results of sensitivity analysis

**Fig. 5:**
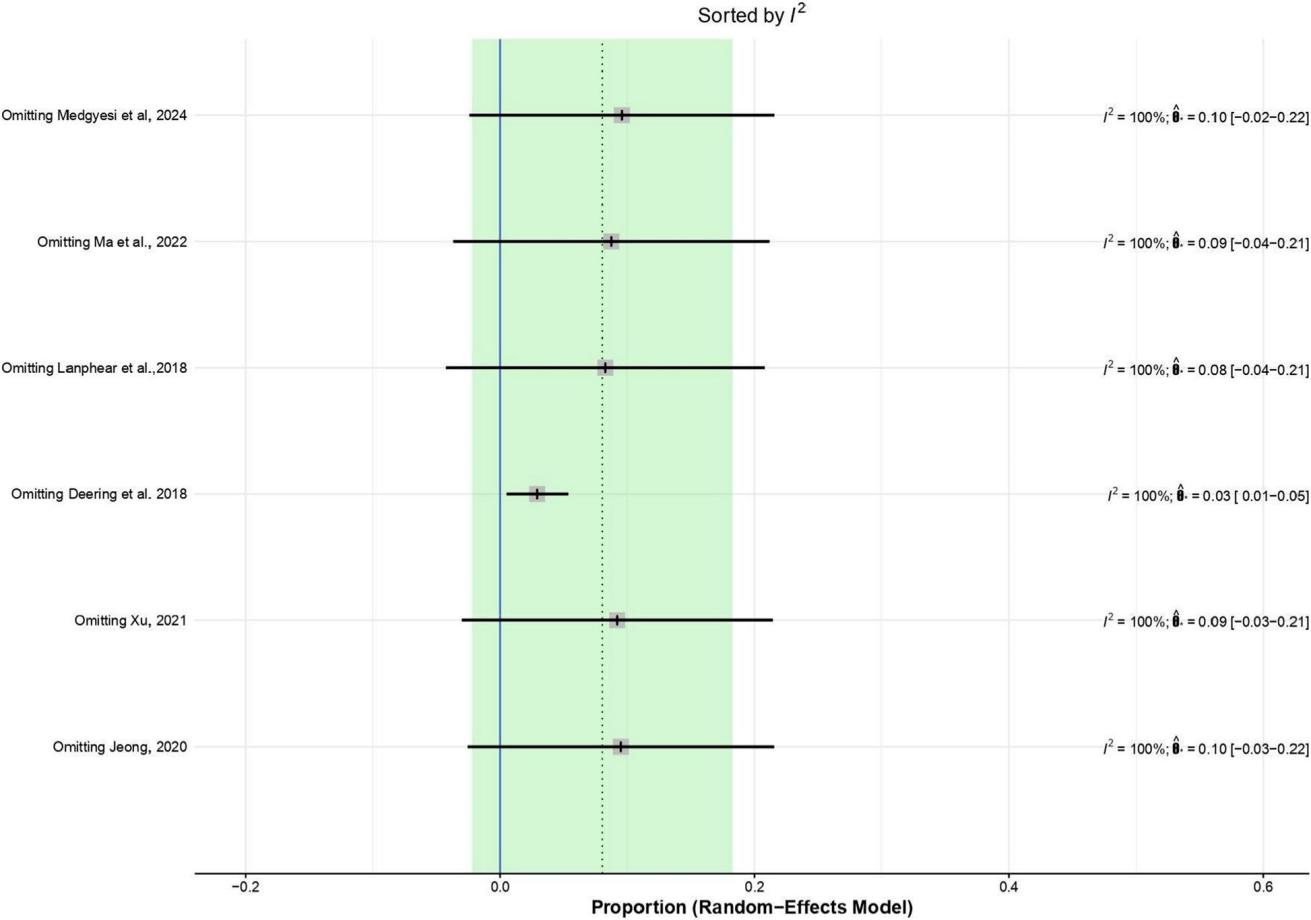
Sensitivity analysis

The leave-one-out sensitivity analysis demonstrated that the overall effect size estimates remained stable when each study was individually excluded, and the confidence intervals of these estimates consistently overlapped zero, indicating no significant change in the overall effect regardless of the study omitted. Despite this stability, the heterogeneity across studies remained extremely high (I2 = 100%) in all iterations, suggesting substantial variability among included studies that was not attributable to any single study. These findings confirm the robustness of the meta-analytic results while highlighting persistent between-study heterogeneity that warrants further investigation. The results are illustrated in Fig. 5.

## Results of meta regression

### CVD

In the regression analyses, age was significantly associated with cardiovascular disease (CVD). Each additional year of age increased the odds of CVD by approximately 0.8% (OR = 1.008, 95% CI: 1.003–1.013, *p* = 0.002; Supplementary Fig. 1a), and the overall model was statistically significant (*p* = 0.002). Body mass index (BMI) showed a non-significant trend toward higher CVD risk, with each one-unit increase in BMI associated with a 2.6% increase in odds (OR = 1.026, 95% CI: 0.993–1.062, *p* = 0.122; Supplementary Fig. 1b). The male-to-female ratio was inversely related to CVD, with higher ratios associated with lower odds, but this was not statistically significant (OR = 0.879, 95% CI: 0.749–1.033, *p* = 0.117; Supplementary Fig. 1c). Overall, age was the only significant independent predictor of CVD in these models.

### CHD

In the meta-regression analyses for coronary heart disease (CHD), age and the male-to-female ratio were significant predictors, whereas body mass index (BMI) was not. Increasing age was associated with a higher risk of CHD, with each additional year corresponding to a 1% increase in the odds (OR = 1.010, 95% CI: 1.008–1.011, *p* < 0.001; Supplementary Fig. 2a). Conversely, a higher male-to-female ratio was associated with a lower risk of CHD, with each unit increase linked to a 27% reduction in odds (OR = 0.728, 95% CI: 0.697–0.759, *p* < 0.001; Supplementary Fig. 2b). By contrast, BMI was not significantly associated with CHD (OR = 1.015, 95% CI: 0.951–1.083, *p* = 0.647; Supplementary Fig. 2c).

### Stroke

In the meta-regression analyses for stroke, age was the only significant predictor. Each additional year of age was associated with a 0.5% increase in the odds of stroke (OR = 1.005, 95% CI: 1.002–1.009, *p* = 0.001; Supplementary Fig. 3a). By contrast, body mass index (BMI) showed a non-significant association with stroke risk, with each unit increase corresponding to a 3.4% rise in odds (OR = 1.034, 95% CI: 0.996–1.072, *p* = 0.077; Supplementary Fig. 2b). Similarly, the male-to-female ratio was not significantly associated with stroke (OR = 0.915, 95% CI: 0.808–1.035, *p* = 0.156; Supplementary Fig. 2c).

**Fig. 6:**
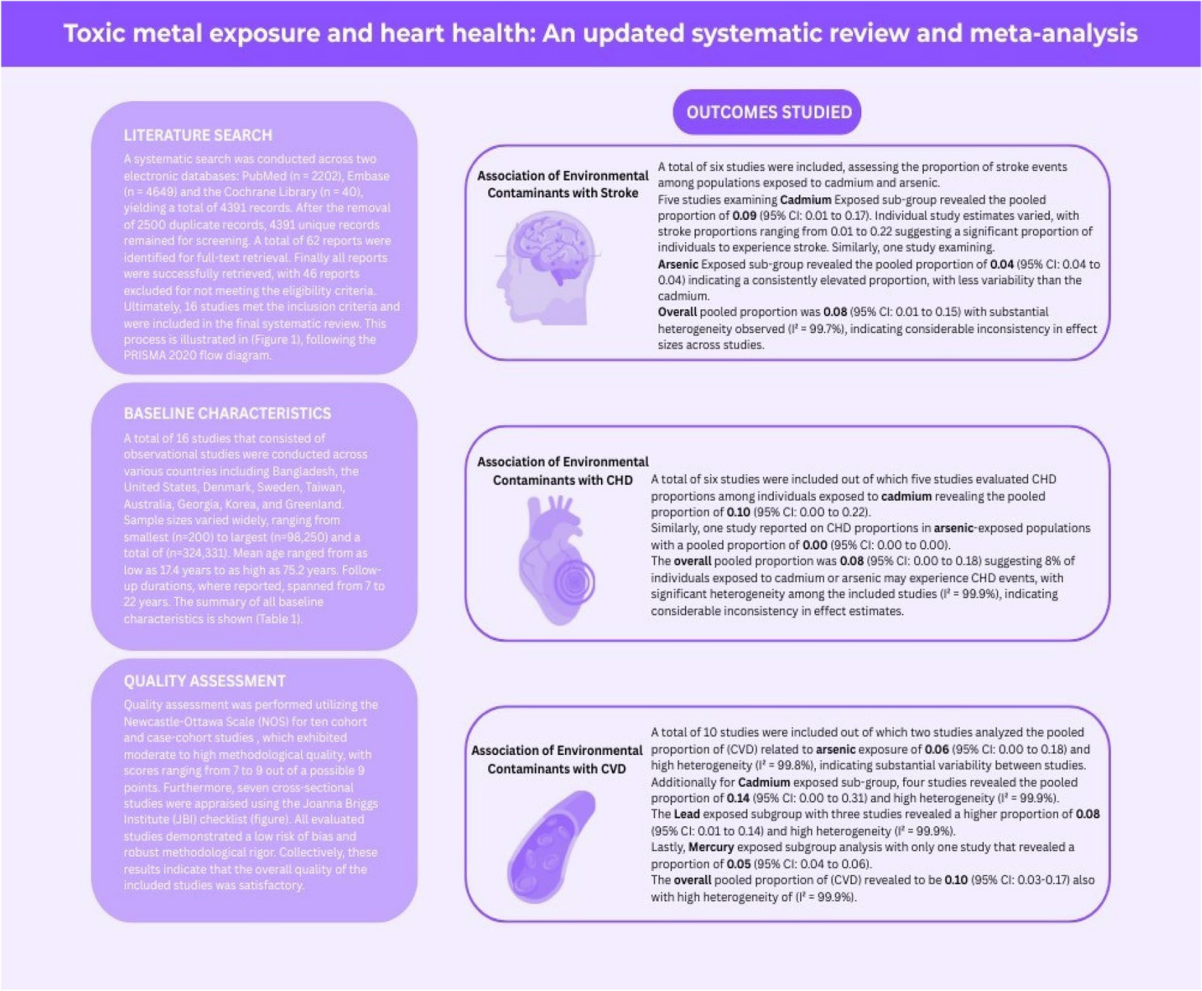
Central illustration

The overall results of this systematic review and meta-analysis are presented in the central illustration below (Fig. 6).

#### Certainty of evidence using GRADE assessment

In pooled analyses of non-randomised studies with follow-up durations ranging from 7 to 22 years, the intervention was associated with a substantially reduced risk of major cardiovascular outcomes. For stroke events (3 studies; 58,253 participants), the hazard ratio (HR) was 0.08 (95% CI, 0.01 to 0.15) with moderate-certainty evidence, indicating a markedly lower risk compared with the control group. Similarly, for coronary heart disease (7 studies; 1,872,157 participants), the HR was 0.08 (95% CI, 0.00 to 0.18), also with moderate-certainty evidence. For overall cardiovascular diseases (14 studies; 1,962,790 participants), the HR was 0.10 (95% CI, 0.03 to 0.17), again demonstrating a strong protective association. Across all outcomes, risk of bias, indirectness, and imprecision were assessed as not serious, but inconsistency was rated very serious. The absolute risk differences could not be calculated due to data limitations, but the relative effect sizes consistently indicated substantial benefit. The summary of findings table is shown in Table 2.

**Table 2:** Summary of Findings (GRADE Assessment)

Certainty assessment							Summary of findings				
**Participants** **(studies)** **Follow-up**	**Risk of bias**	**Inconsistency**	**Indirectness**	**Imprecision**	**Publication bias**	**Overall certainty of evidence**	**Study event rates (%)**		**Relative effect** **(95% CI)**	**Anticipated absolute effects**	
							**With [comparison]**	**With [intervention]**		**Risk with [comparison]**	**Risk difference with [intervention]**
Stroke Events (follow-up: range 7 years to 22 years)											
58,253 (3 non-randomised studies)	not serious	very serious	not serious	not serious		⨁⨁⨁◯ Moderate		58,253 participants	**HR 0.08** (0.01 to 0.15) [Stroke Events]	**Low**	
										0 per 1,000	**– per 1,000** (from – to –)
Coronary Heart Disease (follow-up: range 7 years to 22 years)											
1,872,157 (7 non-randomised studies)	not serious	very serious	not serious	not serious		⨁⨁⨁◯ Moderate		1,872,157 participants	**HR 0.08** (0.00 to 0.18) [Coronary Heart Disease]	**Low**	
										0 per 1,000	**– per 1,000** (from – to –)
Cardiovascular Diseases (follow-up: range 7 years to 22 years)											
1,962,790 (14 non-randomised studies)	not serious	very serious	not serious	not serious		⨁⨁⨁◯ Moderate		1,962,790 participants	**HR 0.10** (0.03 to 0.17) [Cardiovascular Diseases]	**Low**	
										0 per 1,000	**– per 1,000** (from – to –)

*CI* confidence interval, *HR* hazard ratio

## Discussion

Our systematic review and meta-analysis analyzed data from 16 observational studies, which included data from 324,331 participants, to evaluate the association between exposure to environmental toxic metals, specifically cadmium, arsenic, lead, and mercury, and the risk of cardiovascular events, including stroke, cardiovascular disease (CVD), and coronary heart disease (CHD). Overall, our results revealed that individuals exposed to these toxicants exhibited greater chances of cardiovascular events. Notably, cadmium exposure was consistently associated with higher cardiovascular risk across all three outcomes: stroke, CVD, and CHD. Arsenic exposure was also significantly associated with CVD, though based on fewer studies. Lead and mercury exposure show a statistically significant association with CVD; however, neither of them has sufficient evidence to determine any association between ff and CHD individually.

Toxic metals such as cadmium and arsenic induce oxidative stress, systemic inflammation, and endothelial dysfunction, which are key processes in the pathogenesis of atherosclerosis and subsequent cardiovascular events [25, 26]. A common mechanism for metals to cause such disastrous effects involves replacing vital divalent cations [27, 28]. Cadmium especially replaces zinc in numerous enzymes and metalloproteins in the soft tissues, liver, and kidneys, leaving the proteins dysfunctional. This substitution disrupts the function of zinc-dependent enzymes and antioxidant systems, such as superoxide dismutase (SOD), which plays a crucial role in neutralizing reactive oxygen species (ROS). As a result, cadmium exposure leads to increased oxidative stress and lipid peroxidation, damaging vascular endothelial cells and accelerating the development of atherosclerosis, a central mechanism in the pathogenesis of cardiovascular diseases.

Additionally, cadmium may be taken up by several transport proteins, including calcium channels, into the cells of the immune system and infiltrate into vessel walls through cadmium-laden monocytes [29]. The increased production of monocytes and macrophages owing to cadmium may play a significant role in the development and progression of atherosclerosis due to their pivotal roles in the transdifferentiation into foam cells and subsequent necrotic foam cell death, which contributes to endothelial dysfunction. Cadmium-induced endothelial death is the fundamental mechanism by which cadmium causes atherosclerosis, which ultimately leads to CVD. Since it also raises many pro-inflammatory cytokines, including interleukin (IL)−6, IL-8, IL-1β, and tumor necrosis factor-alpha, atherosclerosis is hyperactivated by such an inflammatory response [30–32].

Arsenic is one of the significant clinical concerns regarding elemental toxicities [33]. The endothelium is the vasculature's most vulnerable target for arsenic toxicity. Endothelial dysfunction initiates the development of chronic vascular abnormalities linked to atherosclerosis and cardiovascular illnesses due to an imbalance between vasoconstricting and vasodilating forces [34–36]. Moreover, arsenic induces ROS accumulation by binding to sulfhydryl groups of glutathione, decreasing cardiac glutathione-1 (13) [37]. The reactive oxidation species impair vasodilation by lowering NO and increasing angiotensin II (ANGII) levels. An increase in angiotensin II causes the development of metalloproteinases, such as MMP-2 and MMP-9. It throws off the equilibrium between the endothelium and the underlying vascular smooth muscle, which leads to arterial stiffness [38].

Lead and arsenic are associated with increased levels of soluble adhesion molecules in blood [39]. A 2008 systematic review on lead exposure and cardiovascular health found that population studies demonstrated a link between lead exposure and hypertension and CVD [40]. Lead promotes oxidative stress and limits nitric oxide availability. The study showed that depressed NO availability is paradoxically associated with a marked increase in endothelial NOS (eNOS) and inducible NOS abundance in the kidney and cardiovascular tissues in lead-treated animals. Similarly, because of its strong affinity for sulfhydryl groups, mercury can inactivate a variety of enzymatic processes, amino acids, and sulfur-containing antioxidants, such as N-acetyl L-cysteine, alpha-lipoic acid, and L-glutathione. This can lead to a reduction in antioxidant defense and an increase in oxidative stress [41, 42].

### Metal-specific pathways beyond oxidative stress

Although oxidative stress is a common mechanism across toxic metals, each metal also targets distinct molecular pathways that intensify cellular injury. Cadmium displaces Zinc in superoxide dismutase (SOD), impairing this key antioxidant enzyme and weakening ROS senses [43]. Arsenic promotes aberrant DNA hypermethylation via DNA methyltransferases and upregulates matrix metalloproteinase-9 (MMP-9), thereby driving extracellular matrix degradation and plaque instability [44]. Lead inhibits δ-aminolevulinic acid dehydratase (ALAD), disrupting heme synthesis and increasing pro-oxidant δ-ALA levels [45]. Mercury binds sulfahydral (-SH) groups, depletes glutathione and disrupts thiol-dependant enzymes essential for detoxification. By depleting glutathione (GSH) and impairing GSH-dependent antioxidant enzymes, mercury diminishes cellular detoxification capacity. Moreover, Hg interferes with mitochondrial respiration and calcium signaling, processes essential for vascular and neuronal integrity [46]. A comparative summary of these mechanisms is provided in (Table 3), emphasising that metal- specific molecular interaction complements oxidative stress in promoting vascular and systematic toxicity.

**Table 3:** Metal-specific molecular pathways contributing to toxicity

Metal	Primary Target	Mechanistic Effect
Cadmium (Cd)	Zn site of superoxide dismutase (SOD)	Loss of antioxidant enzyme activity, ↑ROS
Arsenic (As)	DNA methyltransferases; MMP-9	DNA hypermethylation; ECM degradation
Lead (Pb)	δ-aminolevulinic acid dehydratase (ALAD)	Blocked heme synthesis; ↑δ-ALA (pro-oxidant)
Mercury (Hg)	Sulfhydryl (-SH) groups in GSH and enzymes	GSH depletion, mitochondrial dysfunction

The findings of our meta-analysis are primarily consistent with previous literature examining the cardiovascular risks associated with environmental exposure to toxic metals. Prior observational studies and narrative reviews have suggested that chronic exposure to metals like cadmium, arsenic, lead, and mercury contributes to cardiovascular disease development through various mechanisms, including oxidative stress, endothelial dysfunction, and chronic inflammation, as discussed above. For instance, a meta-analysis by Chowdhury et al. [47] reported a significant positive association between toxic metal exposure and cardiovascular mortality, supporting the elevated pooled proportions observed in our analysis. Similarly, studies have linked arsenic exposure, mainly through contaminated drinking water, with an increased incidence of both stroke and coronary heart disease, findings that align with our pooled estimates despite being derived from fewer included studies [48–50].

Lead has also been extensively studied regarding hypertension and CVD. Several large-scale studies and reviews have found that lead exposure is associated with increased all-cause and cardiovascular mortality [51–53]. Our findings provide a more precise assessment of the burden of cardiovascular events among exposed groups by validating and quantifying these correlations using pooled proportions.

In contrast, while mercury exposure has been historically linked to cardiovascular toxicity in animal and experimental models, epidemiological evidence in humans remains limited and inconsistent [54, 55]. Our analysis includes only one mercury-related study, which shows a significant association with CVD [ref of this study]. This suggests that this area remains under-investigated in the human population and requires further exploration.

Significantly, our meta-analysis concentrated on pooled proportions, which give an absolute measure of event occurrence. At the same time, numerous previous studies reported effect estimates such as hazard ratios (HRs), odds ratios (ORs), or relative risks (RRs). Calculating the actual burden of CVD outcomes owing to toxic metal exposure offers a valuable perspective that would be easier to include in public health planning.

This study has several notable strengths. As an updated systematic review and meta-analysis, it provides a comprehensive and current synthesis of the available evidence, enhancing the reliability of its conclusions. All the current evidence has been pooled together, which creates this paper to be the ultimate evidence for our topic. The inclusion of a large, diverse population of over 325,000 individuals from multiple countries and several different states increases the generalizability of the findings across different healthcare settings and populations. Furthermore, the data were derived from original observational studies, including both cohort and cross-sectional designs, which reflect real-world scenarios and strengthen the external validity of the results. By integrating recent studies and applying rigorous inclusion criteria and meta-analytic methods, this review offers an updated and globally relevant perspective on our topic. Researchers and reviewers are provided with up-to-date information and insights from around the literature.

While strengths are there to be appreciated, this review has several limitations as well that should be considered when interpreting the findings. First, the inclusion was limited to English-language articles, which may have introduced language bias and excluded potentially relevant studies published in other languages. Second, although all included studies were assessed to have a low risk of bias, the absence of randomized controlled trials limits the ability to infer causality. The most important factor of all was. Substantial heterogeneity across all outcomes (I2 ≈ 99%), likely resulting from differences in study design, population characteristics, geographic settings, and outcome definitions. Despite a sensitivity analysis by the leave-one-out method being conducted, the value for heterogeneity remained high throughout. Additionally, most included studies were observational, comprising cohorts and cross-sectional studies, which, while informative for identifying associations, are limited in controlling for confounding factors. Lastly, although the studies were conducted in a diverse set of countries, including Bangladesh, the United States, Denmark, Sweden, Taiwan, Australia, Georgia, Korea, and Greenland, regions such as Africa and South America were underrepresented, which may limit the global applicability of the findings.

The findings of this study carry critical implications for public health at both national and global levels. The consistent association between exposure to environmental toxic metals and increased risk of cardiovascular and stroke-related outcomes underscores the importance of addressing environmental determinants of non-communicable diseases. Toxic metals such as lead, cadmium, arsenic, and mercury are often present in air, water, soil, and food, especially in industrialized and developing regions. Their widespread presence and potential for bioaccumulation mean that large segments of the population may be at risk, often unknowingly.

Given the observed health impacts, public health authorities need to implement and enforce stricter environmental regulations aimed at reducing metal contamination. Additionally, public health campaigns are needed to raise awareness about sources of exposure and ways to minimize risk, especially in vulnerable communities. These findings support a shift toward a more comprehensive model of cardiovascular disease prevention that includes environmental health as a core component.

While this review highlights a clear association between toxic metal exposure, several gaps in the literature remain that should be addressed in future research. First, most of the included studies were observational, which limits the ability to draw firm causal conclusions. Future investigations should prioritize longitudinal cohort studies and, where ethical and feasible, interventional trials to better establish causality. Second, there is a need for more geographically diverse data. Expanding research efforts into these underrepresented regions would enhance the global applicability of findings and ensure that local environmental and socioeconomic factors are adequately captured. Furthermore, future studies should aim to standardize methods of exposure assessment and outcome measurement to reduce heterogeneity and improve comparability across studies. Ultimately, advancing this area of research will require interdisciplinary collaboration across environmental science, epidemiology, toxicology, and public health policy to develop effective strategies for risk reduction and health risks.

## Conclusion

Exposure to environmental toxic metals, including cadmium, arsenic, lead, and mercury, is associated with an increased risk of cardiovascular events such as stroke, coronary heart disease, and overall cardiovascular disease. Cadmium consistently showed a strong link across all outcomes, while arsenic and lead were also significantly associated with increased risk. Evidence regarding mercury was limited but suggested a potential relationship with cardiovascular disease that warrants further investigation.

Although this review included a large and diverse population, and the findings were drawn from recent studies, several limitations must be acknowledged, particularly the high heterogeneity across studies and the reliance on observational data. Despite these limitations, the results emphasize the importance of addressing environmental exposures in efforts to prevent cardiovascular diseases. There is a clear need for more research in underrepresented regions and for studies that can better clarify causal relationships.

## Supplementary Information

Supplementary Material 1

## Data Availability

The datasets generated and/or analyzed during the current study are available in the manuscript or as supplementary file.
