# Supplementary material for: Toxic heavy metal exposure and heart health: a systematic review and meta-analysis of 324,331 patients
Source: BMC Cardiovasc Disord. 2025 Nov 7;25:792. doi: 10.1186/s12872-025-05248-9 (PMC12595707; doi:10.1186/s12872-025-05248-9)
Supplement: Supplementary file 1 — Supplementary Material 1 [file 12872_2025_5248_MOESM1_ESM.docx]

| PubMed | **(cadmium OR arsenic OR lead OR mercury) AND (environmental exposure OR metal exposure OR toxic metal exposure OR heavy metal exposure) AND (cardiovascular disease OR CVD OR coronary heart disease OR CHD OR stroke OR ischemic heart disease OR myocardial infarction) AND (risk OR association OR odds OR hazard OR relative risk OR risk ratio)** | 2202 |
| --- | --- | --- |
| Embase | (**'cadmium'**/exp OR **cadmium** OR **'arsenic'**/exp OR **arsenic** OR **'lead'**/exp OR **lead** OR **'mercury'**/exp OR **mercury**) AND (**'environmental exposure'**/exp OR **'environmental exposure'** OR (**environmental** AND (**'exposure'**/exp OR **exposure**)) OR **'metal exposure'**/exp OR **'metal exposure'** OR ((**'metal'**/exp OR **metal**) AND (**'exposure'**/exp OR **exposure**)) OR **'toxic metal exposure'** OR (**toxic** AND (**'metal'**/exp OR **metal**) AND (**'exposure'**/exp OR **exposure**)) OR **'heavy metal exposure'**/exp OR **'heavy metal exposure'** OR (**heavy** AND (**'metal'**/exp OR **metal**) AND (**'exposure'**/exp OR **exposure**))) AND (**'cardiovascular disease'**/exp OR **'cardiovascular disease'** OR ((**'cardiovascular'**/exp OR **cardiovascular**) AND (**'disease'**/exp OR **disease**)) OR **cvd** OR **'coronary heart disease'**/exp OR **'coronary heart disease'** OR (**coronary** AND (**'heart'**/exp OR **heart**) AND (**'disease'**/exp OR **disease**)) OR **chd** OR **'stroke'**/exp OR **stroke** OR **'ischemic heart disease'**/exp OR **'ischemic heart disease'** OR (**ischemic** AND (**'heart'**/exp OR **heart**) AND (**'disease'**/exp OR **disease**)) OR **'myocardial infarction'**/exp OR **'myocardial infarction'** OR (**myocardial** AND (**'infarction'**/exp OR **infarction**))) AND (**'risk'**/exp OR **risk** OR **'association'**/exp OR **association** OR **odds** OR **'hazard'**/exp OR **hazard** OR **'relative risk'**/exp OR **'relative risk'** OR ((**'relative'**/exp OR **relative**) AND (**'risk'**/exp OR **risk**)) OR **'risk ratio'**/exp OR **'risk ratio'** OR ((**'risk'**/exp OR **risk**) AND (**'ratio'**/exp OR **ratio**))) | 4649 |
| Cochrane | **(cadmium OR arsenic OR lead OR mercury) AND (environmental exposure OR metal exposure OR toxic metal exposure OR heavy metal exposure) AND (cardiovascular disease OR CVD OR coronary heart disease OR CHD OR stroke OR ischemic heart disease OR myocardial infarction) AND (risk OR association OR odds OR hazard OR relative risk OR risk ratio)** | 40 |

Table S1: Search strategies

| Forkan et al., 2021 | Risk of bias: Low - Methodological quality: High -- The study meets all 8 JBI criteria, indicating a robust and well-conducted cross-sectional analysis. |
| --- | --- |
| Chen, 2018. | Total NOS Score: 8/9 stars , This study demonstrates high methodological quality and low risk of bias across all domains of the Newcastle-Ottawa Scale. |
| Stine, 2018 | Total NOS Score: 8/9 stars , This study demonstrates high methodological quality and low risk of bias across all domains of the Newcastle-Ottawa Scale. |
| Lanphear et al.,2018 | Total NOS Score: 9/9 stars , This study demonstrates high methodological quality and low risk of bias across all domains of the Newcastle-Ottawa Scale. |
| Deering et al. 2018 | Total NOS Score: 9/9 stars , This study demonstrates high methodological quality and low risk of bias across all domains of the Newcastle-Ottawa Scale. |
| Ma et al., 2022 | Risk of bias: Low - Methodological quality: High -- The study meets all 8 JBI criteria, indicating a robust and well-conducted cross-sectional analysis. |
| Harari et al,. 2019 | Risk of bias: Low _ Methodological quality: High _ The study meets all 8 JBI criteria, indicating a robust and well-conducted cross-sectional analysis. |
| Kuo et al,. 2021 | Total NOS Score: 9/9 stars. This study demonstrates high methodological quality and low risk of bias across all domains of the Newcastle-Ottawa Scale. |
| Jeong, 2020 | Risk of bias: Low - Methodological quality: High - The study meets all 8 JBI criteria, indicating a robust and well-conducted cross-sectional analysis. |
| Lin, 2020 | Risk of bias: Low _ Methodological quality: High _ The study meets all 8 JBI criteria, indicating a strong, well-conducted cross-sectional design. |
| Annette, 2018 | Total NOS Score: 9/9 stars , High-quality cohort study, scoring the maximum on the Newcastle-Ottawa Scale. |
| Medgyesi et al, 2024 | Total NOS Score: 9/9 stars , High-quality cohort rating under the Newcastle-Ottawa Scale. |
| Ma et al., 2022 | Risk of bias: Low - Methodological quality: High -- The study meets all 8 JBI criteria, indicating a robust and well-conducted cross-sectional analysis. |

Table S2: Quality assessment table
